# Neuroprotective effects of miR-142-5p downregulation against isoflurane-induced neurological impairment

**DOI:** 10.1186/s13000-020-00978-0

**Published:** 2020-06-06

**Authors:** Cuili Xie, Hongyue Wang, Yu Zhang, Yanhua Wei

**Affiliations:** 1 Department of Anesthesiology, Jining No. 1 People’s Hospital, No. 6, Jiankang Road, Jining, Shandong 272011 People’s Republic of China; 2 Jining Medical University, Jining, Shandong 272011 People’s Republic of China

**Keywords:** miR-142-5p, Isoflurane, Neurological impairment, Viability, Apoptosis

## Abstract

**Background:**

Isoflurane can lead to neuron damage to the developing brain, resulting in learning and memory disability. The aim of this study was to investigate the role of miR-142-5p on isoflurane-induced neurological impairment.

**Methods:**

The Morris water maze (MWM) test was performed to evaluate spatial learning and memory of rats. The expression level of miR-142-5p was measured using qRT-PCR. MTT assay was used to calculate the viability of hippocampal neuronal cells. The cell apoptosis was analyzed using Flow cytometric assay.

**Results:**

Isoflurane treatment led to the increase of neurological function score and escape latency, and the reduction of time spent in the original quadrant in rats. The expression level of miR-142-5p was increased significantly in isoflurane-treated rats. MiR-142-5p downregulation protected against isoflurane-induced neurological impairment, which was reflected by the decrease of neurological function score and escape latency, and the increase of time spent in the original quadrant. In vitro, downregulation of miR-142-5p alleviated isoflurane-induced neuron cell viability inhibition, and relieved isoflurane-induced cell apoptosis.

**Conclusions:**

MiR-142-5p downregulation plays a neuroprotective role in protecting against isoflurane-induced neurological impairment through regulating neuron cell viability and apoptosis. It provides a theoretical basis for the investigation of the mechanism underlying the effect on isoflurane-induced neurological impairment.

## Introduction

Isoflurane is a common inhaled anesthetic during surgical procedure [1]. It is reported that the use of isoflurane can lead to neuron damage on the developing brain, resulting in learning, writing and reading disability [2, 3]. Animal studies have shown that isoflurane application contributes to neuron cell apoptosis, which in turn leads to cognitive impairment of rats [4, 5]. Notably, early isoflurane exposure may result in persistent learning deficits and cognitive dysfunction in children and rodents [6]. However, the pathogenesis of isoflurane-induced neurological impairment is not fully understood.

MicroRNAs (miRNAs) are a group of small noncoding RNA, with the length of 22–25 nucleotides [7]. The abnormal expression of miRNA has been identified to be involved in the regulation of diverse cellular processes [8, 9]. In recent years, miRNAs have shown potential effect for the management of neurological diseases, including neuron injury induced by anesthesia [10–12]. For example, Wu et al. have suggested that downregulation of miR-448 plays an important role in improving isoflurane-induced learning and memory impairment through regulating neuron apoptosis [13]. Another study in neonatal rats has also reported that the application of sevoflurane elevates the level of miR-96, which promotes hippocampal neuron apoptosis and weakens the learning and memory performance of the rats [14]. Recently, miR-142-5p is reported to play a crucial role in neuron injury, and involved in the regulation of cellular survival [15]. However, its role in isoflurane-induced neurological impairment has not been investigated.

In the current study, the effect of miR-142-5p on isoflurane-induced neurological impairment was investigated in isoflurane-treated rats. We further explored the role of miR-142-5p in neuron cell viability and apoptosis.

## Materials and methods

### Animal preparation and grouping

The current study was approved by the Ethics Committee of the Experimental Animal Center of Jining No. 1 People’s Hospital. All animals were treated according to the Guide for the Care and Use of Laboratory Animals of the Institute for Laboratory Animal Research.

Sprague-Dawley (SD) rat pups (15–20 g) of postnatal day 7 (P7) were purchased from Changzhou Cavens Laboratory Animal Co. Ltd. All the rats were raised following the guidelines. The rats were randomly divided into four groups with 12 rats in each group: 1) control group: rats received regular air inhalation for 6 h; 2) isoflurane group: rats were exposed to isoflurane (1.5%) for 6 h [16]; 3) antagomir NC group: rats were given 2 nmol miR-142-5p antagomir NC by lateral cerebroventricular injection, and 30 min later, exposed to 1.5% isoflurane for 6 h; 4) miR-142-5p antagomir group: rats were given 2 nmol miR-142-5p antagomir by lateral cerebroventricular injection, and 30 min later exposed to 1.5% isoflurane for 6 h. After isoflurane treatment, six rats in each group were randomly selected and euthanized by decapitation, and the hippocampi tissues were collected for further experiments. And the remaining rats were used for following neurological scoring and Morris Water maze evaluation.

### Neurological function evaluation

6 neonatal rats of each group were fed until day 14, and the behavior and motor changes of the experimental rats were evaluated using the 20-point neuron score, as previously reported [16]. The behavior evaluation included the response to the response to and circling of nociceptive stimuli, postural and walking reflexes, extremity tonus, performance in a smooth climbing platform, and consciousness. A score of 0 points indicates unimpaired neurological function, and a score of 20 points indicates the most severe neurological dysfunction.

### Morris water maze test

6 neonatal rats (P14) of each group were fed until day 14, then the Morris water maze (MWM) test was performed to evaluate spatial learning and memory of rats as previously described [17]. In brief, the rats were forced to finish a swim test for 4 consecutive days in a circular water pool that was separated into four quadrants of equal size. The water pool was 80 cm in deep and 100 cm in diameter, with a 30 cm depth of water and a hidden circular platform 2 cm below the water surface. The swimming paths of rats were recorded and analyzed using VideoMot software version 2.4.50923 (TSE Systems GmbH, Bad Homburg, Germany) regarding the following parameters: escape latency and time in the original quadrant. Rats were placed in the maze from four random points of the tank and were allowed to find the hidden platform for 2 min. If this was not achieved, the rat was gently placed on the platform and left for 20 s.

### Cell culture and transfection

As described in previous research, primary hippocampal cells were collected from P0 newborn rats and cultured [18]. Cells were cultured in Neurobasal medium (Invitrogen; Thermo Fisher Scientific, Inc., Waltham, MA, USA) in a humidified incubator with 5% CO_2_ at 37 °C. Once a week, replace one-third of the medium with fresh Neurobasal medium. After incubation, microtubule-associated protein 2 (MAP 2) was used for the identification of primary hippocampal cells according to the previous evidence [19].

The cells were divided into four groups: 1) Untreated negative control group (NC group); 2) isoflurane group (cells were treated with 2% isoflurane for 6 h); 3) isoflurane + inhibitor group (cells were treated with isoflurane and transfected with miR-142-5p inhibitor NC); 4) isoflurane +miR-inhibitor group (cells were treated with isoflurane and transfected with miR-142-5p inhibitor). MiR-142-5p inhibitor and its negative control (inhibitor NC) were provided by Gene-Pharma (Shanghai, China). Cells were plated into 96-well plates with a density of 1 × 10^5^ cells/well, and cultured for 24 h. Then cells transfection was performed using Lipofectamine 2000 (Invitrogen, Carlsbad, CA, USA) according to the manufacturer’s protocols.

### RNA extraction and quantitative real-time polymerase chain reaction (qRT-PCR)

Total RNA was extracted by using Trizol reagent (Invitrogen, Carlsbad, CA, USA) according to the manufacturer’s protocol. cDNA was synthesized by using TaqMan miRNA reverse transcription kit (Applied Biosystems, Foster City, CA, USA). The qRT-PCR assay was performed using a SYBR Green I Real-Time PCR Kit (GenePharma, Shanghai, China) to detect the gene expression. The following thermocycling conditions were used for the PCR: Initial denaturation at 95 °C for 5 min; 30 cycles of 95 °C for 30 s, 60 °C for 30 s and 72 °C for 20 s; and a final extension at 72 °C for 10 min. After amplification, a melting curve was generated to evaluate the specificity of PCR products at the end of each PCR cycle. And the amplification of only one product in qRT-PCR was confirmed by a melting curve analysis. U6 was used as an internal control, and the relative expression of miR-142-5p was calculated using the comparative delta CT (2^−ΔΔCt^) method. The primers used were as follows: miR-142-5p forward, 5′- GGGCAUAAAGUAGAAAGC-3′ and reverse, 5′-CTCAACTGGTGTCGTGGA-3′; U6 forward, 5′-CTCGCTTCGGCAGCACA-3′ and reverse, 5′-AACGCTTCACGAATTTGCGT-3′.

### MTT assay

MTT assay was used to calculate the viability of hippocampal neuronal cells. 48 h post-transfection, the stably transfected cells were plated into a 96-well plate with a density of 1 × 10^4^ cells/well. At 0 h, 24 h, 48 h, 72 h after cell seeding, the cells were stained with 20 μl of MTT (5 mg/ml; Sigma-Aldrich; Merck, Darmstadt, Germany) respectively at each time point. After incubation for another 4 h, dimethyl sulfoxide (DMSO) (Sigma-Aldrich; Merck) was added into each well. The absorbance was measured at 490 nm using a microplate reader.

### Flow cytometric assay

The cell apoptosis of hippocampal neuronal cells was analyzed using the Annexin V-FITC Fluorescence Microscopy kit (BD Biosciences, San Jose, CA, USA). Cells of each group were harvest and centrifuged to collect 5 × 10^5^ neurons, followed by fixation with 3.7% formaldehyde for 15 min at room temperature and permeabilization with 0.1% Triton X­100 for 5 min at 37 °C. Then cells were washed with PBS buffer for three times, and resuspended in the 1 × Binding Buffer. Subsequently, the cells were mixed with 5 μl Annexin V­fluorescein isothiocyanate and propidium iodide. After incubation for 10 min at room temperature, the apoptotic rates were measured by using a FACScan flow cytometer (BD Biosciences). The cell apoptotic rates were counted as the sum of both early and late apoptotic rates.

### Statistical analysis

All experiments were performed independently three times. All the data analyses were performed using GraphPad Prism 5.0 software (GraphPad Software, Inc., USA). The differences between groups were compared using student’s *t*-test or one-way ANOVA analysis. *P* < 0.05 was considered to be statistically significant.

## Results

### Effects of isoflurane on neurological impairment in rats

**Fig. 1:**
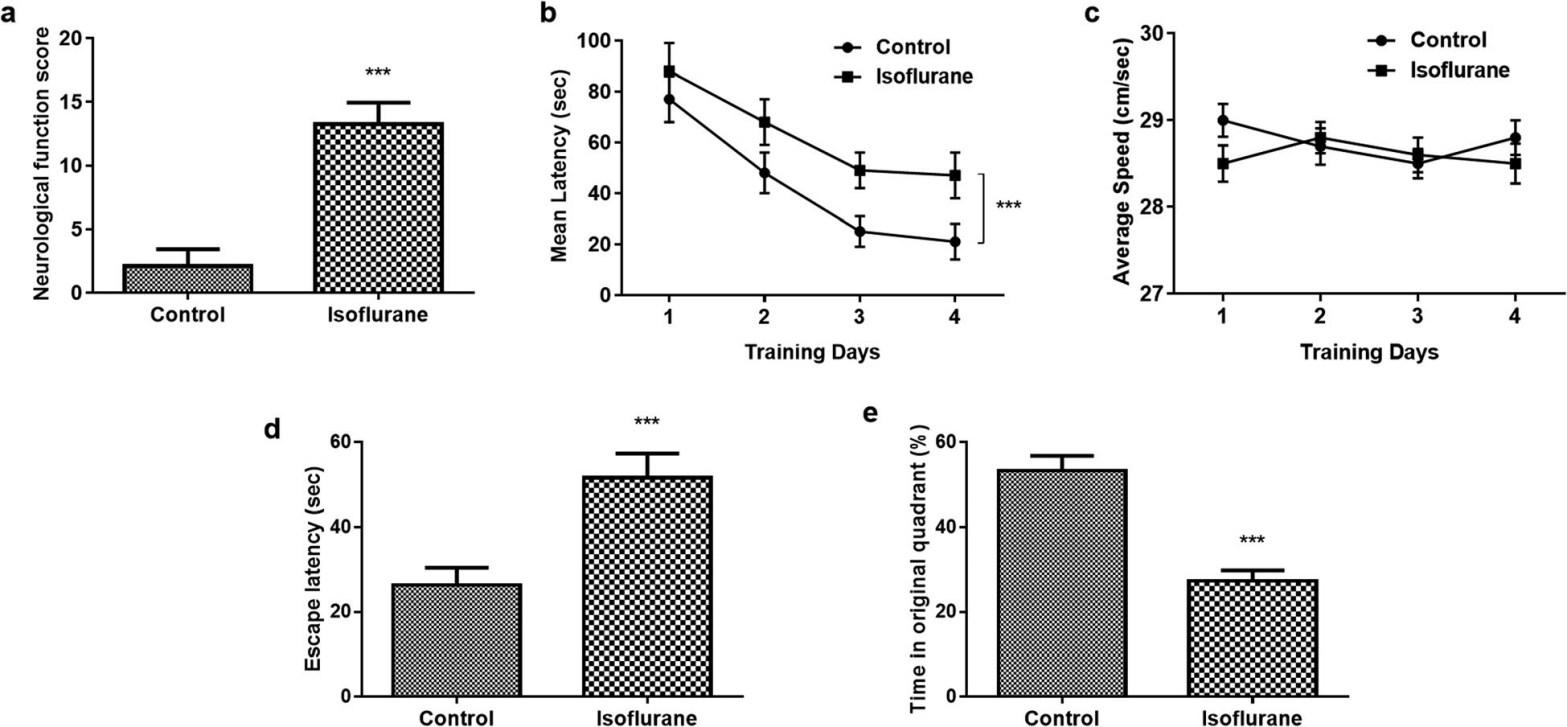
Effects of isoflurane on neurological impairment in rats. **a** The neurological function score increased significantly in isoflurane treated rats compared with the control group. **b** During the spatial acquisition training time, the time required to locate the platform was significantly affected by isoflurane treatment compared with the control group. **c** Isoflurane treatment did not influence the swimming speed of rats during the spatial acquisition training time. **d-e** A probe trial was conducted to assess reference memory at the end of learning. Isoflurane treatment significantly enhanced the escape latency and reduced the time in the original quadrant of rats compared with the control group. *** *P* < 0.001

Neurological examination and morris water maze test were performed to calculate the effect of isoflurane on neurological impairment in rats. As shown in Fig. 1a, it was found that the neurological function score increased significantly in isoflurane treated rats compared with the control group (*P* < 0.001). Additionally, the Morris water maze test results suggested that during the spatial acquisition training time, the time required to locate the platform was significantly affected by isoflurane treatment compared with the control group (Fig. 1b). However, as Fig. 1c suggested, isoflurane treatment did not influence the swimming speed of rats. Furthermore, a probe trial was conducted to assess reference memory at the end of learning. It was found that the escape latency was significantly enhanced in isoflurane group compared with the control group, whereas the time in the original quadrant was significantly reduced by isoflurane treatment (*P* < 0.001, Fig. 1d-e). These results indicated that isoflurane treatment can lead to impaired learning and memory, and the isoflurane-induced neuron injury model in rats was successfully established.

### The expression level of miR-142-5p in rats treated with isoflurane

**Fig. 2:**
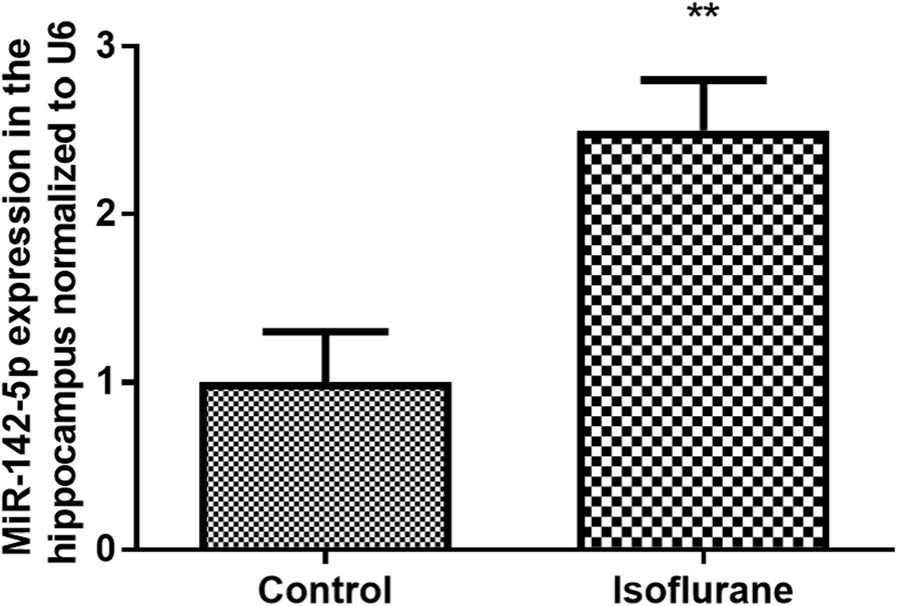
The expression level of miR-142-5p in rats treated with isoflurane. The expression level of miR-142-5p was increased significantly in the hippocampus of isoflurane-treated rats compared with the control group. ** *P* < 0.01

The expression level of miR-142-5p was detected using qRT-PCR. The results suggested that the expression level of miR-142-5p was increased significantly in the hippocampus of isoflurane-treated rats compared with the control group (*P* < 0.01, Fig. 2). It was concluded that the high expression of miR-142-5p expression might be associated with the neuron injury caused by isoflurane treatment.

### The effect of miR-142-5p on isoflurane-induced neurological impairment

**Fig. 3:**
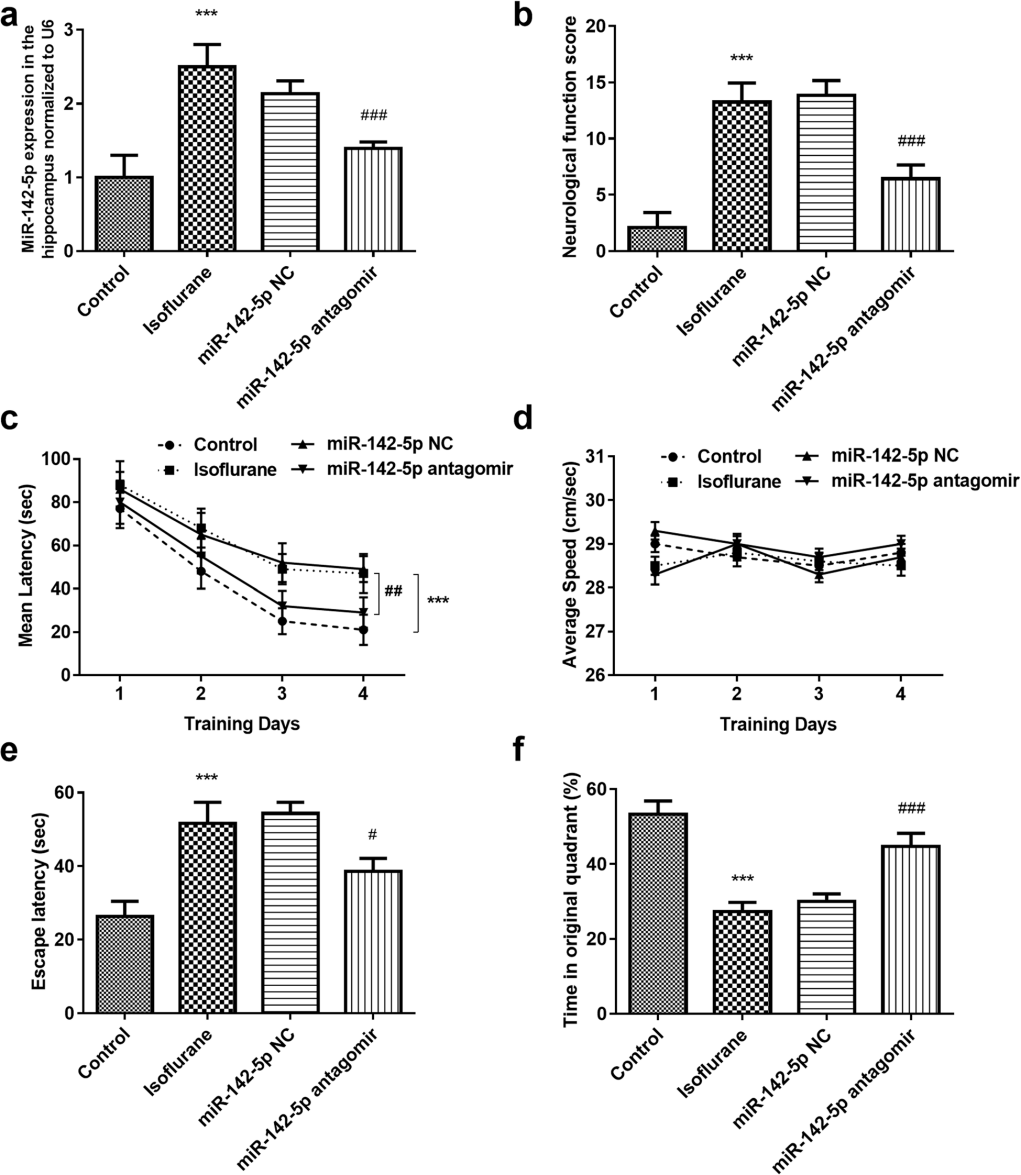
The effect of miR-142-5p on isoflurane-induced neurological impairment. **a** The increasing trend of miR-142-5p expression induced by isoflurane treatment was significantly attenuated by the downregulation of miR-142-5p. **b** MiR-142-5p downregulation significantly reduced the neurological function score which was increased by isoflurane treatment. **c-d** MiR-142-5p downregulation significantly alleviated the influence of isoflurane on the latency time of rats during the spatial acquisition training time, but showed no significant influence on the swimming speed. **e-f** MiR-142-5p downregulation reversed the effects of isoflurane treatment on the escape latency and the time in the original quadrant at the end of learning. *** *P* < 0.001, compared with control group; ^#^*P* < 0.05, ^###^*P* < 0.001, compared with isoflurane group

To explore the effect of miR-142-5p on isoflurane-induced neurological impairment in rats, the expression level of miR-142-5p in rats was regulated via miR-142-5p antagomir injection. As shown in Fig. 3a, the increasing trend of miR-142-5p expression induced by isoflurane treatment was significantly attenuated by the downregulation of miR-142-5p (*P* < 0.001). The neurological examination results demonstrated that miR-142-5p downregulation significantly reduced the neurological function score which was increased by isoflurane treatment (*P* < 0.001, Fig. 3b). Additionally, the Morris water maze test results revealed that miR-142-5p downregulation significantly alleviated the influence of isoflurane on the latency time of rats during the spatial acquisition training time, but showed no significant influence on the swimming speed (Fig. 3c-d). Furthermore, a probe trial was conducted to assess reference memory at the end of learning. It was found that miR-142-5p downregulation reversed the effects of isoflurane treatment on the escape latency and the time in the original quadrant in rats at the end of learning. (*P* < 0.05, Fig. 3e-f).

### The effect of miR-142-5p on hippocampal neuron cell viability and apoptosis

**Fig. 4:**
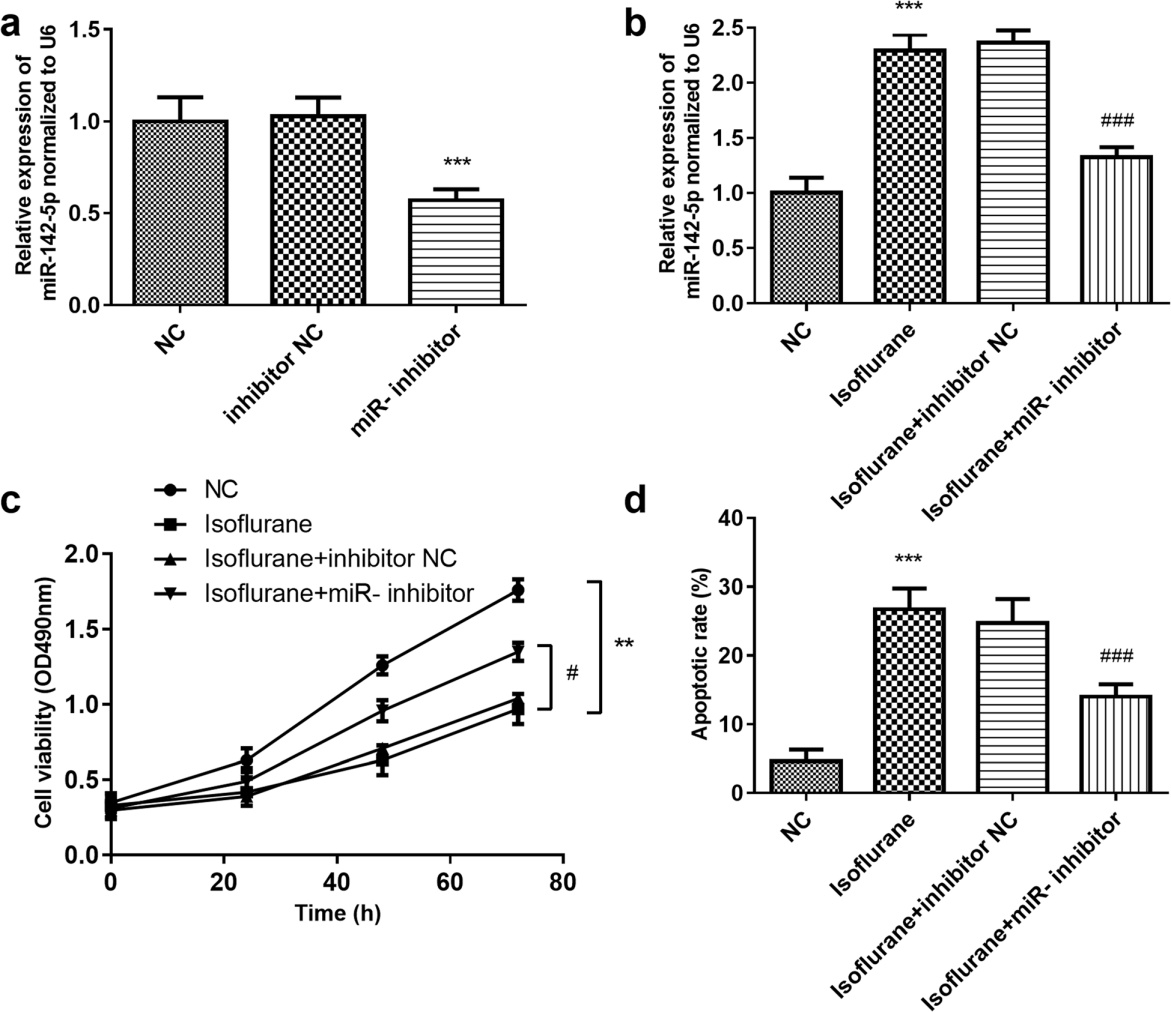
The effect of miR-142-5p on hippocampal neuron cell viability and apoptosis. **a** MiR-142-5p inhibitor transfection significantly reduced miR-142-5p level. **b** Isoflurane treatment significantly increased the expression level of miR-142-5p in hippocampal neurons, but miR-142-5p inhibitor transfection significantly reversed the effect. **c** MTT assay results suggested that isoflurane treatment inhibited the cell viability significantly, which was alleviated by miR-142-5p downregulation. **d** Flow cytometry assay results demonstrated that isoflurane treatment promoted cell apoptosis, while miR-142-5p downregulation relieved isoflurane-induced cell apoptosis. *** *P* < 0.001, compared with control group; ^#^*P* < 0.05, ^###^*P* < 0.001, compared with isoflurane group

We further investigated the effects of miR-142-5p on cell viability and cell apoptosis of hippocampal neurons. qRT- PCR results indicated that miR-142-5p inhibitor transfection significantly reduced miR-142-5p level (Fig. 4a). Additionally, as shown in Fig. 4b, isoflurane treatment significantly increased the expression level of miR-142-5p in hippocampal neurons, but miR-142-5p inhibitor transfection significantly reversed the effect (*P* < 0.001). Additionally, the MTT assay and flow cytometry assay results demonstrated that isoflurane treatment inhibited the cell viability significantly (*P* < 0.01, Fig. 4c), while the cell apoptosis was remarkably promoted by isoflurane treatment (*P* < 0.001, Fig. 4d). Furthermore, it was found that transfection with miR-142-5p inhibitor significantly alleviated isoflurane-induced cell viability inhibition, and relieved isoflurane-induced cell apoptosis (*P* < 0.05, Fig. 4c-d).

## Discussion

Isoflurane, an inhalation anesthetic, is considered to be safe and effective in pediatric anesthesia [20]. As a result of the rapid induction, early recovery, low impact on liver and kidney function, and stable hemodynamics, isoflurane has been widely used in clinical [21, 22]. In recent years, with the wide application of anesthetic drugs, the anesthetic complications have been increased annually [23]. Notably, the role of anesthetic drugs in the central nervous system, especially in the ability of learning and memory for infants and children, has been widely reported [24, 25]. Recently, there is an increasing focus on neuron injury-induced by isoflurane.

In the present study, the isoflurane-induced neurological impairment injury model in rats was established. It was detected that isoflurane treatment led to the impaired ability of learning and memory in rats, which was reflected by the increase of neurological function score and escape latency, and the reduction of time spent in original quadrant during the Morris water maze test. These results indicated that the isoflurane-induced neurological impairment model in rats was successfully established. Additionally, in hippocampal neurons from newborn rats, it was noted that isoflurane treatment inhibited the cell viability and promoted the cell apoptosis, which might be the underlying mechanism of the damaging effect of isoflurane on learning and memory. Consistently, Zhu et al. reported that isoflurane treatment significantly impaired the object recognition and reversal learning in young rats, and increased number of cell death of progenitors or neurons in the hippocampus was also detected, suggesting the cognitive deficits induced by isoflurane occurred in a clearly age-dependent manner [26]. Thus, in our present study, the rat pups of 7 days old were used for the construction of isoflurane-treated models. Additionally, another study in aged rats also suggested that the spatial memory can be impaired for 2 weeks after general anesthesia with isoflurane in aged rats [27]. All evidence determined the damaging effect of isoflurane application on the nervous system.

In the past years, the crucial role of miRNAs in multiple types of human diseases have been reported, including neurological diseases. The dysregulation of miRNAs has been reported to be involved in the regulation of biological process and pathological processes [28, 29]. Notably, the involvement of miRNAs in anesthetic-induced neuron injury has been proposed in recent researches. A study in isoflurane-treated rats reported that miR-448 knockdown suppressed neuron apoptosis, and further participated in the regulation of isoflurane-induced learning and memory impairment [13]. Another study about sevoflurane-induced cognitive dysfunction revealed that the addition of miR-96 promoted cell apoptosis induced by sevoflurane and exacerbated the cognitive function impairment of the rats [14]. The present results found that isoflurane treatment significantly increased the expression level of miR-142-5p in the hippocampus of rats, suggesting the potential role of miR-142-5p in the neuron injury caused by isoflurane application. Consistently, in a study about cerebral ischemia/reperfusion (I/R) injury, miR-142-5p was proved to be induced in hippocampal neurons by oxygen-glucose deprivation and reoxygenation (OGD/R) treatment, and inhibition of miR-142-5p attenuated OGD/R induced neuron injury [15]. Another study in Alzheimer’s disease (AD) also determined that miR-142-5p is highly expressed in AD patients, indicating the involvement in the pathological process of AD [30]. In the current study, we further investigated the effect of miR-142-5p on learning and memory impairment using the knockdown method. As expected, miR-142-5p downregulation protected against isoflurane-induced neurological impairment, which was reflected by the decrease of neurological function score and escape latency, and the increase of time spent in original quadrant during the Morris water maze test. These results demonstrated the important role of miR-142-5p in neurological impairment, and the downregulation of miR-142-5p may play neuroprotective effects on isoflurane-induced neurological impairment.

MiR-142-5p has been reported to be related to cell viability and apoptosis. In pancreatic cancer, miR-142-5p was reported to play the role of cancer suppressor through regulating pancreatic cancer cell proliferation and apoptosis [31]. Another study by Yang et al. suggested that miR-142-5p functions as a growth promotive miRNA and plays an important role in neurogenic differentiation of adipose-derived stem cells (ADSCs) [32]. In the present study, the isoflurane-induced neurological impairment model in neuron cell was constructed, and the expression level of miR-142-5p was regulated by cell transfection. It was found that downregulation of miR-142-5p alleviated isoflurane-induced cell viability inhibition, and relieved isoflurane-induced cell apoptosis, which might be the underlying mechanism of the involvement of miR-142-5p in isoflurane-induced neurological impairment. However, in the current study only neurons were analyzed, and the effect of isoflurane on other cells, such as astrocytes, microglia is known, which is needed to be further investigated. Further experimental studies are required to explore the underlying mechanism in depth.

Taken together, the current study demonstrated that miR-142-5p downregulation plays a neuroprotective role in protecting against isoflurane-induced neurological impairment through regulating neuron cell viability and apoptosis. It provides a theoretical basis for the investigation of the mechanism underlying the effect on isoflurane-induced neurological impairment.

## Data Availability

The datasets used and/or analyzed during the current study are available from the corresponding author on reasonable request.

## Abbreviations

MWM: Morris water maze
miRNAs: microRNAs
SD: Sprague-Dawley
P7: Postnatal day 7
NC group: Negative control group
inhibitor NC: Inhibitor negative control
qRT-PCR: Quantitative real-time polymerase chain reaction
DMSO: Dimethyl sulfoxide
OGD/R: Xygen-glucose deprivation and reoxygenation
AD: Alzheimer’s disease
ADSCs: Adipose-derived stem cells
